# Digestive behavior and gut microbiota responses of *Glehnia littoralis* polysaccharide–Iron complexes: Influence of polysaccharide molecular weight

**DOI:** 10.1016/j.fochx.2026.103777

**Published:** 2026-03-20

**Authors:** Xuan Hu, Xia Liu, Yu Zhang, Yanli Yu, Quanfang Zhang, Xueyan Gao, Wei Liu

**Affiliations:** a Institute of Food & Nutrition Science and Technology, Shandong Academy of Agricultural Sciences, Jinan 250100, China; b Medical Science and Technology Innovation Center, Shandong First Medical University & Shandong Academy of Medical Sciences, Jinan 250117, China; c Sports & Medicine Integration Research Center (SMIRC) Capital University of Physical Education and Sports, Beijing 100191, China; d Institute of Resources and Environment, Dezhou Academy of Agricultural Sciences, Dezhou 253000, China

**Keywords:** *Glehnia littoralis*, Polysaccharide–iron complexes, *In vitro* digestion, *In vitro* fermentation, Gut microbiota

## Abstract

*Glehnia littoralis* polysaccharides (GLPs) were fractionated by graded ethanol precipitation to yield fractions with different molecular weights, which were then coordinated with iron to construct GLPs–Iron complexes exhibiting distinct iron-binding ratios. Structural characterization confirmed that iron coordination occurred predominantly through hydroxyl and carboxyl groups without disrupting the polysaccharide backbone. *In vitro* digestion revealed that maximum gastric iron release decreased with increasing polysaccharide molecular weight (an approximately 1.96-fold difference between the lowest and highest fractions), while all complexes maintained low and stable intestinal release. *In vitro* fermentation demonstrated that native GLPs promoted the growth of *Lactobacillus*, while GLPs–Iron complexes, unlike free iron, were associated with an increased relative abundance of *Bacteroides* species involved in complex carbohydrate degradation. These results indicate polysaccharide–iron conjugation stabilizes iron and modulates microbial responses, highlighting a promising strategy for developing iron fortification systems with improved digestive stability and targeted microbiota interactions.

## Highlights

• GLPs were fractionated and chelated with iron into stable, molecular weight–defined complexes.
• Iron coordinated *via* hydroxyl and carboxyl groups without polysaccharide backbone disruption.
• Iron release was molecular weight–dependent, with low-weight complexes showing higher gastric release.
• GLPs promoted Lactobacillus, whereas GLPs–Iron complexes enriched beneficial Bacteroides.

## 1 Introduction

Iron deficiency remains one of the most prevalent micronutrient deficiencies worldwide, particularly affecting children, women of reproductive age, and populations in developing regions (Lopez et al., 2016; Pasricha et al., 2021). Food iron fortification is widely adopted to improve dietary iron intake; however, the application of conventional iron fortificants is often limited by poor physicochemical stability, low solubility, and undesirable reactions during gastrointestinal digestion, which compromise their effectiveness in food systems (Detzel & Wieser, 2015; Naktinienė et al., 2021).

Dietary iron is commonly delivered in the form of inorganic iron salts, such as ferrous sulfate, ferrous succinate, and ferrous gluconate. Despite their high iron content, these compounds are prone to redox reactions that may induce lipid peroxidation and gastrointestinal irritation (Kaye et al., 2008; Qi & Yue, 2020). In recent years, polysaccharide–iron complexes have emerged as promising food-grade iron carriers due to their improved coordination stability, enhanced gastrointestinal tolerance, and favorable bioaccessibility (Wang et al., 2020).

Only a limited fraction of orally administered iron from fortified supplements is absorbed. For instance, ferrous sulfate absorption averages only ∼16% in iron-deficient women (Stoffel et al., 2017), while elemental iron powders used in flour fortification exhibit absorption rates as low as <2–3% (Zimmermann et al., 2010). Consequently, the majority of ingested iron remains unabsorbed and enters the colon, where it inevitably interacts with and modulates the gut microbiota (Zimmermann et al., 2010). Previous studies have demonstrated that excessive iron intake can induce significant alterations in gut microbial composition. Specifically, iron fortification in nutritional supplements was shown to disturb gut microbial homeostasis, leading to a reduction in beneficial bacterial populations and a greater persistence of potentially pathogenic species (Cuisiniere et al., 2025; Das et al., 2020; Fang et al., 2018). Therefore, the potential impact of iron supplements themselves on the gut microbiota should not be overlooked when considering iron supplementation.

Polysaccharides used as iron ligands are often endowed with intrinsic prebiotic properties, serving as fermentable substrates that modulate gut microbial composition and metabolic activity (Ho Do et al., 2021). However, the combined effects of polysaccharides and coordinated iron on microbial fermentation behavior are more complex and remain insufficiently understood. *Glehnia littoralis* Fr. Schmidt ex Miq. is a traditional edible plant widely consumed in East Asia, and polysaccharides derived from its dried root represent major bioactive components with established functional food potential (Chen et al., 2021; Du et al., 2019). Recent studies have shown that polysaccharide–iron complexes derived from *Glehnia littoralis* exhibit enhanced functional properties compared with polysaccharides alone (Yongshuai et al., 2018).

Given that the molecular weight of dietary polysaccharides is a key determinant of their structural organization, gastrointestinal behavior, and fermentability, it was hypothesized that the molecular weight of *Glehnia littoralis* polysaccharides (GLPs) determines the coordination density of the resulting polysaccharide–iron complexes, thereby influencing iron release behavior during gastrointestinal digestion and consequently modulating microbial responses during *in vitro* colonic fermentation. To test this hypothesis, GLPs fractions were prepared *via* graded ethanol precipitation and coordinated with iron. Using an *in vitro* gastrointestinal digestion and fecal fermentation model, the effects of these polysaccharide fractions and their iron complexes on iron release, microbial community structure were systematically evaluated. This study provides insights into the rational design of plant-derived, microbiota-friendly iron fortificants for food applications.

## 2 Materials and methods

### 2.1 Materials

*Glehnia littoralis* dried root was purchased from Chifeng, Inner Mongolia, China, ground into powder, and passed through an 80-mesh sieve prior to use. Botanical authentication of the plant material was formally conducted by Professor Fuhua Bian from Yantai University (Yantai, Shandong, China). A voucher specimen (No. GL-20250309) was deposited at Yantai University for future reference. Ferric chloride (FeCl₃) was obtained from Tianjin Damao Chemical Reagent Factory (Tianjin, China). Hydroxylamine hydrochloride and phenol were purchased from Shanghai Macklin Biochemical Co., Ltd. (Shanghai, China). The Bradford protein assay kit was supplied by Beyotime Biotechnology (Shanghai, China). All other chemical reagents used in this study were of analytical grade and purchased from Sinopharm Chemical Reagent Co., Ltd. (Shanghai, China).

### 2.2 Preparation of GLPs fractions

*Glehnia littoralis* dried root powder was defatted with petroleum ether and anhydrous ethanol, followed by hot-water extraction (80 °C, 2 h × 3). After centrifugation and concentration, polysaccharides were precipitated with ethanol, deproteinized using the Sevag method, and further fractionated by stepwise ethanol precipitation (40%, 60%, and 80%) to obtain P40, P60, and P80, representing approximately 76%, 13%, and 11% of the total mass (wt%), respectively.

### 2.3 Preparation of GLPs–Iron complex

The GLP*s*–Iron complex was prepared with minor modifications to a previously reported method (L. Wang et al., 2020). FeCl₃ solution was added dropwise to a GLPs solution containing sodium citrate under continuous stirring, followed by pH adjustment to 8.0 and incubation at 60 °C for 4 h. After centrifugation, the supernatant was dialyzed (MWCO 500 Da) against distilled water and freeze-dried to obtain polysaccharide–iron complexes. Complexes derived from P40, P60, and P80 were denoted as P40Fe, P60Fe, and P80Fe, respectively.

### 2.4 Characterization of GLPs–Iron complexes

#### 2.4.1 Iron content

Iron content was determined according to a previously reported method with minor modifications (J. Wang et al., 2015). Briefly, dried GLPs–Iron complexes (10 mg) were treated with 1 M HCl to dissociate the complexes, followed by colorimetric determination using hydroxylamine hydrochloride and 1,10-phenanthroline.

#### 2.4.2 Scanning Electron microscopy (SEM)

The surface morphology of GLPs fractions and their corresponding iron complexes was examined using SEM analysis. Lyophilized samples were ground, mounted on conductive stubs, gold-coated, and observed at various magnifications.

#### 2.4.3 X-ray diffraction (XRD) analysis

XRD patterns of the GLPs and their corresponding iron complexes were recorded using an X-ray diffractometer (SmartLab SE, Rigaku, Tokyo, Japan). The diffraction data were collected over a 2θ range of 10°–70° to evaluate the crystalline structure of the samples.

#### 2.4.4 UV–vis spectroscopy

UV–Vis spectra of the samples were collected at room temperature in the range of 200–800 nm using a UV–Vis–NIR spectrophotometer (Cary 5000, Agilent Technologies, California, USA) to assess electronic transitions associated with iron complexation.

#### 2.4.5 Fourier transform infrared (FT-IR) spectroscopy

FT-IR spectra were recorded using an FT-IR spectrometer (INVENIO-S, Bruker, Ettlingen, Germany) with the KBr pellet method. Samples were scanned over the wavenumber range of 4000–350 cm^−1^ to identify functional groups involved in polysaccharide–iron interactions.

#### 2.4.6 X-ray photoelectron spectroscopy (XPS) analysis

XPS analysis was performed on a Thermo Scientific K-Alpha spectrometer with a monochromatic Al Kα source. High-resolution spectra (C 1 s, O 1 s, Fe 2p) were calibrated to the adventitious C 1 s peak (284.8 eV) and curve-fitted using Thermo Avantage software.

### 2.5 *In vitro* simulated gastrointestinal digestion and determination of Iron release from GLPs–Iron complexes

*In vitro* digestion was performed according to the standardized INFOGEST protocol with minor modifications (Brodkorb et al., 2019). Electrolyte stock solutions were prepared according to the INFOGEST formulation, and the simulated gastric fluid (SGF) and simulated intestinal fluid (SIF) were prepared to the final electrolyte concentrations listed in Supplementary Table S1. All digestion fluids were preheated to 37 °C before use, and CaCl₂ solution was added immediately prior to digestion to a*v*oid precipitation during storage. Enzyme activities were verified according to standard assay procedures to ensure batch-to-batch consistency. Simulated gastric digestion was conducted by mixing the sample solution (3 mg/mL) with preheated SGF (1:1, *v*/v), adjusting the pH to 2.0, and adding pepsin (2000 U/mL) and gastric lipase (60 U/mL), followed by incubation at 37 °C for 2 h. Aliquots were collected at predetermined time points and immediately cooled on ice.

For the intestinal phase, gastric chyme was mixed with preheated SIF, supplemented with pancreatin (100 U/mL based on trypsin activity) and bile salts (10 mM), and adjusted to pH 7.0. The mixture was incubated at 37 °C with shaking, and aliquots were collected at selected time points. Samples were filtered (0.22 μm) and ultrafiltered (1 kDa), and the released Fe was quantified using the 1,10-phenanthroline method. Digestion blank controls (buffer replacing the sample) and enzyme blank controls (digestive fluids without sample) were performed in parallel to correct for background signals.

### 2.6 *In vitro* fermentation

*In vitro* fermentation was performed with minor modifications to a previously reported method (Zhang et al., 2024). All animal experimental procedures were appro*v*ed by the Institutional Animal Care and Use Committee (IACUC) of Shandong First Medical University (Approval No. W202508290860) and conducted in accordance with the Chinese National Standard *Laboratory Animal—Guideline for Ethical Review of Animal Welfare* (GB/T 35892–2018). Cecal contents were collected from fifteen 8-week-old male C57BL/6 mice maintained on a standard laboratory diet and housed under specific pathogen-free (SPF) conditions in individually ventilated cages. Prior to sample collection, mice were anesthetized with isoflurane and euthanized by cervical dislocation. The samples were pooled to obtain a representative microbial inoculum, diluted (1:10, *w*/*v*) in sterile PBS, homogenized, and filtered to prepare a fecal slurry. An aliquot of the slurry (1 mL) was inoculated into 9 mL of anaerobic culture medium supplemented with P40, P60, P80, or their corresponding polysaccharide–iron complexes (P40Fe, P60Fe, and P80Fe) at a final concentration of 5 mg/mL. An equivalent concentration of FeSO₄ was added to the medium and designated as the Fe group. Inulin and substrate-free medium were used as the positive and blank controls, respectively. Fermentation was conducted anaerobically at 37 °C for 24 h. After 24 h of incubation, the samples were collected, centrifuged, and stored at −80 °C for subsequent microbial analyses.

### 2.7 Gut microbiota analysis

Gut microbiota composition was analyzed by 16S rRNA gene sequencing. Microbial DNA was extracted from the *in vitro* simulated fermentation products as described in Section 2.6. The V3–V4 regions of the 16S rRNA gene were amplified with primers 338F/806R and sequenced on an Illumina MiSeq platform (paired-end). Sequence processing and taxonomic analysis were performed using QIIME. Differentially abundant taxa were identified by LEfSe analysis (LDA > 4.0, *P* < 0.05). Sequencing data were deposited in the NCBI under accession No. PRJNA1395796.

### 2.8 Statistics

Statistical analyses were performed using GraphPad Prism 8.0 (GraphPad Software, USA). Group differences were evaluated by one-way ANOVA followed by Tukey's *post hoc* test. Data are presented as mean ± SD, with P < 0.05 considered statistically significant.

## 3 Results and discussion

### 3.1 Preparation of GLPs–Iron Complexes

**Fig. 1:**
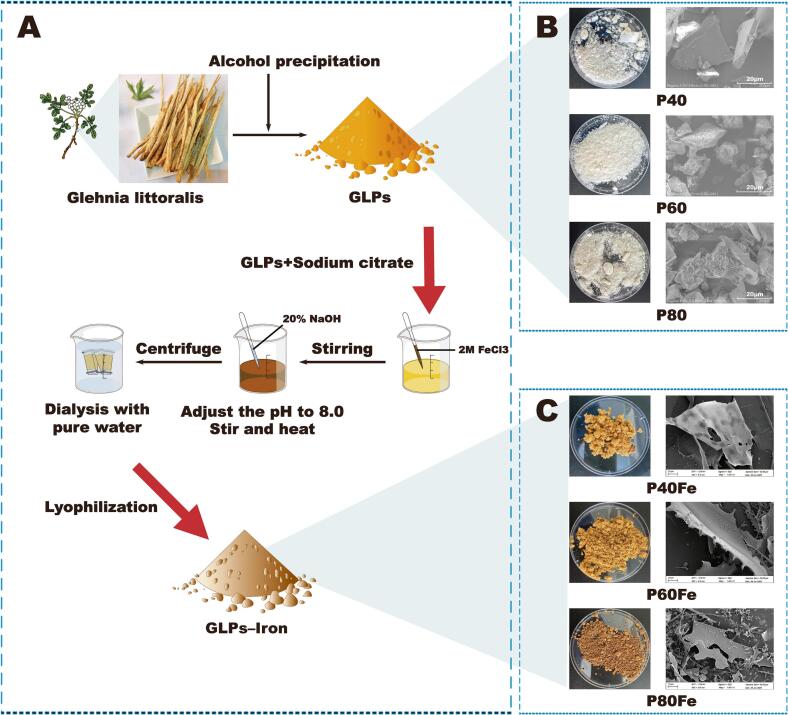
(A) Schematic illustration of the preparation of *Glehnia littoralis* polysaccharides (GLPs) fractions by graded ethanol precipitation and subsequent iron coordination to obtain GLPs–Iron complexes. (B) Photographs and scanning electron microscopy (SEM) images of GLPs fractions (P40, P60, and P80) and (C) the corresponding GLPs–Iron complexes (P40Fe, P60Fe, and P80Fe).

The preparation of GLPs fractions with different molecular weights and their corresponding iron complexes is shown in Fig. 1A. Polysaccharides were fractionated by graded ethanol precipitation at 40%, 60%, and 80% (designated P40, P60, and P80) and subsequently chelated with iron to obtain P40Fe, P60Fe, and P80Fe. As summarized in Table 1 and Fig. 1, purified polysaccharide fractions with distinct molecular characteristics were successfully obtained, and iron chelation yielded dark reddish-brown complexes, indicating effective polysaccharide–iron coordination.

**Table 1:** Physicochemical properties of GLPs fractions and their iron complexes.

	Protein (%)	Uronic acid (%)	Reducing sugar (%)	Fe (%)	Mw (g/mol)
P40	4.36 ± 1.15	5.72 ± 0.07	2.67 ± 0.14	0	26,956
P60	3.03 ± 2.32	5.22 ± 0.05	8.3 ± 0.1	0	6304
P80	5.36 ± 1.04	3.2 ± 0.02	23.05 ± 0.21	0	1352
P40Fe	2.91 ± 0.6	–	–	21.14 ± 0.29	–
P60Fe	1.91 ± 1.28	–	–	24.88 ± 0.26	–
P80Fe	2.05 ± 0.47	–	–	35.69 ± 0.35	–

The molecular weights of the polysaccharide fractions decreased with increasing ethanol concentration, following the order P40 > P60 > P80, with Mw values of 26,956, 6304, and 1352 g/mol, respectively. This distribution is consistent with the principle of graded ethanol precipitation, whereby higher-molecular-weight polysaccharides preferentially precipitate at lower ethanol concentrations (Bian et al., 2010). Scanning electron microscopy revealed pronounced molecular weight–dependent morphological differences (Fig. 1B). P40 exhibited relatively dense, plate-like structures with smooth surfaces, whereas P60 and P80 showed progressively smaller, more fragmented, and irregular morphologies. After iron complexation, all samples displayed more compact and aggregated structures with increased surface roughness, suggesting iron-induced intermolecular crosslinking and structural rearrangement (T. Liu et al., 2019). Notably, P80Fe exhibited the most densely packed morphology.

Iron was effectively incorporated into all polysaccharide fractions, while no iron was detected in the native polysaccharides. The iron contents increased significantly with decreasing molecular weight, reaching 21.14 ± 0.29% (P40Fe), 24.88 ± 0.26% (P60Fe), and 35.69 ± 0.35% (P80Fe). This trend indicates that lower-molecular-weight polysaccharides provide more accessible coordination sites, likely due to reduced steric hindrance and greater exposure of functional groups, consistent with previous reports on polysaccharide–metal complexes (Cui et al., 2018).

Overall, graded ethanol precipitation effectively generated GLPs fractions with distinct molecular and structural features, which governed their iron-binding capacity. The resulting GLPs–Iron complexes with tunable iron contents provide a well-defined system for subsequent studies on digestion behavior and gut microbiota modulation.

### 3.2 Structural and physicochemical characterization of GLPs–Iron complexes

**Fig. 2:**
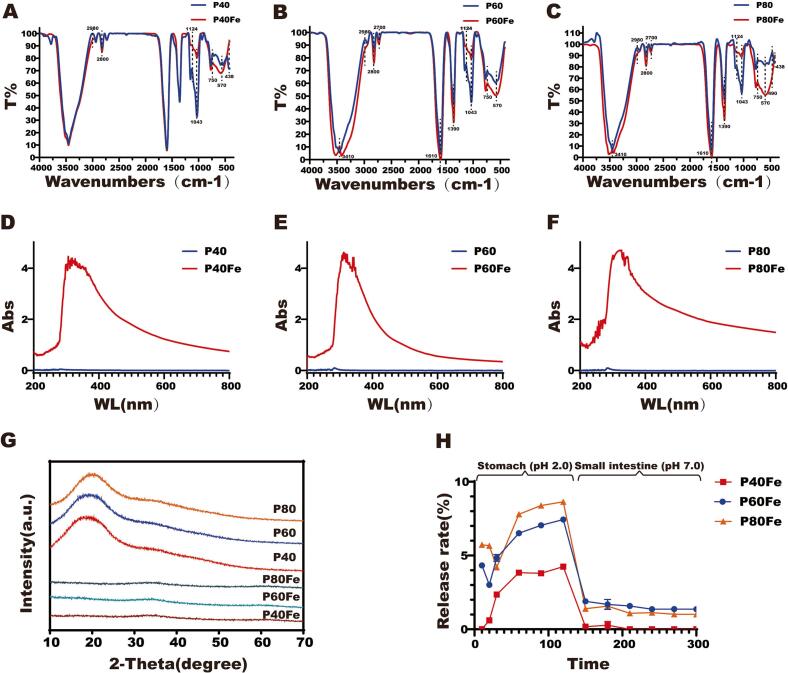
FTIR spectra of GLPs fractions and their corresponding iron complexes: (A) P40 and P40Fe; (B) P60 and P60Fe; (C) P80 and P80Fe. UV–Vis spectra of GLPs fractions and their corresponding iron complexes: (D) P40 and P40Fe; (E) P60 and P60Fe; (F) P80 and P80Fe. (G) X-ray diffraction (XRD) patterns of native GLPs and GLPs–Iron complexes. (H) Iron release profiles of GLPs–Iron complexes during *in vitro* simulated gastrointestinal digestion.

FTIR spectroscopy revealed pronounced structural differences between the native polysaccharide fractions (P40, P60, and P80) and their corresponding iron complexes (Fig. 2A-2C). Compared with the native polysaccharides, the characteristic absorption bands at approximately 3410, 1610, 1390, 1124, and 1043 cm^−1^ were markedly weakened or broadened upon iron complexation, indicating that hydroxyl and carboxyl groups served as the primary coordination sites for Fe(III) (Jiang et al., 2025; J. Wang et al., 2015). Similar attenuation of O—H— and C=O-related bands has been widely reported for plant-derived polysaccharide–iron complexes and is generally attributed to the formation of metal–oxygen coordination bonds (M. Liu et al., 2024). In addition, a new absorption band appeared in the low-wavenumber region at around 490 cm^−1^, which can be attributed to Fe—O vibrations. This feature suggests the formation of iron–oxygen coordination structures within the polysaccharide matrix, consistent with the incorporation of iron species in polysaccharide–iron complexes prepared *via* FeCl₃-mediated coordination (Jing et al., 2022).

UV–Vis spectroscopy further confirmed the formation of polysaccharide–iron complexes (Fig. 2D-2F). Native polysaccharides showed nearly featureless spectra between 200 and 800 nm, consistent with the lack of intrinsic chromophores and indicating high sample purity (Novo & Edgar, 2024). In contrast, the GLPs–Iron complexes exhibited markedly enhanced absorption in the UV region (200–400 nm), which is commonly attributed to ligand-to-metal charge transfer interactions between Fe^3+^ and electron-donating hydroxyl and carboxyl groups in the polysaccharide chains. Similar UV absorption enhancement has been reported for plant-derived polysaccharide–iron complexes and is considered characteristic of successful metal coordination (Zhou et al., 2022).

Consistent with the FTIR results, X-ray diffraction (XRD) analysis further demonstrated distinct structural differences between the native polysaccharides and their iron complexes (Fig. 2G). The diffraction patterns of the polysaccharide samples exhibited a broad halo centered at approximately 20°, which is characteristic of amorphous polysaccharides and reflects the absence of long-range crystalline order. In contrast, the broad diffraction feature at ∼20° disappeared in the GLPs–Iron complexes, suggesting that iron coordination substantially altered the original hydrogen-bonding interactions within the polysaccharide matrix(M. Liu et al., 2024). This observation is in good agreement with the FTIR results, which suggested coordination-induced structural rearrangement rather than backbone degradation.

Surface characterization *via* XPS provided further evidence for Fe—O coordination (Fig. S1). The complexes exhibited surface iron contents of 2.45%–3.17% alongside a noticeable downshift in O 1 s binding energies (*e.g.*, 532.39 to 532.07 eV for the P40/P40Fe transition). Such spectral shifts suggest electron donation from oxygen to Fe^3+^, supporting chemical coordination rather than simple physical entrapment (Wei et al., 2024).

The combined XRD, UV–Vis, FTIR and XPS analyses consistently demonstrate that Fe^3+^ coordination induces significant structural and electronic modifications without disrupting the fundamental polysaccharide backbone, thereby confirming the successful construction of stable polysaccharide–iron complexes.

### 3.3 Iron release behavior of GLPs–iron complexes during *In Vitro* simulated gastrointestinal digestion

Based on *in vitro* simulated digestion (Fig. 2H), polysaccharide–iron complexes exhibited molecular weight–dependent iron release behaviors in both gastric (pH 2.0) and intestinal (pH 7.0) phases. During the gastric phase, iron release followed a first-order kinetic model (Table S2). Both the maximum release (*F*_*max*_) and the area under the curve (AUC) increased with decreasing molecular weight (P80Fe > P60Fe > P40Fe), suggesting progressively weaker Fe^3+^ chelation in the lower-molecular-weight polysaccharide fractions under acidic conditions. In contrast, upon transition to the intestinal phase, iron release from all complexes rapidly decreased and remained low and stable, markedly differing from inorganic iron salts that readily precipitate at neutral pH (Wei et al., 2024). The polysaccharide matrix effectively stabilized Fe^3+^ through coordination with hydroxyl and carboxyl groups, with P60Fe and P80Fe maintaining slightly higher releasability than P40Fe. Overall, these results demonstrate that polysaccharide fractionation enables modulation of iron release, yielding complexes with limited gastric dissociation and sustained intestinal stability (L. Wang et al., 2020).

### 3.4 Effects of GLPs–iron complexes on gut microbiota composition and fermentation profiles

**Fig. 3:**
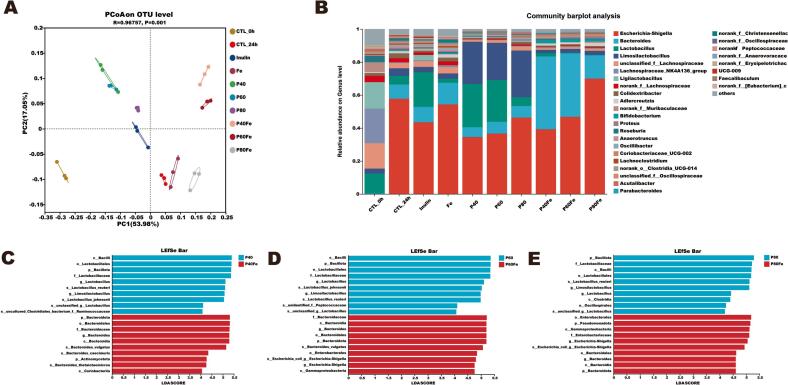
(A) Beta-diversity analysis of gut microbiota based on principal coordinates analysis (PCoA). (B) Relative abundance of the dominant gut microbial genera after 24 h of *in vitro* fermentation. LEfSe analysis showing differentially abundant taxa between native GLPs fractions and their corresponding iron complexes: (C) P40 *vs.* P40Fe; (D) P60 *vs.* P60Fe; (E) P80 *vs.* P80Fe.

The effects of different GLPs fractions and their iron complexes on gut microbiota during *in vitro* fermentation were further evaluated. Beta-diversity analysis based on OTU-level PCoA revealed a clear separation among treatment groups (Fig. 3A), with PC1 and PC2 explaining 53.98% and 17.05% of the total variance, respectively. ANOSIM analysis confirmed a significant difference in overall microbial structure (*R* = 0.96757, *P* = 0.001). After 24 h of fermentation, the control group (CTL_24h) was clearly separated from all GLPs- and GLPs–Iron complexes-treated groups, indicating that both GLPs and their iron complexes markedly reshaped the microbial community. Native polysaccharide fractions (P40, P60, and P80) clustered closely and were positioned near the inulin group, suggesting comparable prebiotic-like modulation. In contrast, the corresponding GLPs–Iron complexes (P40Fe, P60Fe, and P80Fe) were distinctly separated from their native counterparts along PC1, indicating that iron coordination substantially altered fermentation-driven microbial responses. Free iron (Fe group) formed a separate cluster, demonstrating a distinct impact of iron alone on community composition. Moreover, differences among P40Fe, P60Fe, and P80Fe suggest that polysaccharide molecular characteristics modulate the extent to which iron influences microbial assembly.

Genus-level community barplot analysis further supported these observations (Fig. 3B). Compared with the initial control (CTL_0h), CTL_24h exhibited a marked increase in *Escherichia–Shigella* (Yin et al., 2025), indicating the expansion of potentially opportunistic taxa in the absence of functional substrates. A similar enrichment was observed in the Fe group, suggesting that free iron alone may favor the proliferation of this genus under *in vitro* fermentation conditions. In contrast, native GLPs significantly reduced the relative abundance of *Escherichia–Shigella* while promoting putatively beneficial genera, mainly *Lactobacillus* (Mejía-Caballero & Marco, 2025; Sanders et al., 2019) and *Limosilactobacillus* (Abuqwider et al., 2022). Distinct from the native GLPs, GLPs–Iron complexes exhibited unique compositional profiles. In both the P40Fe and P60Fe groups, suppression of *Escherichia–Shigella* was accompanied by a pronounced increase in *Bacteroides*, a genus specialized in complex carbohydrate utilization. Although P80Fe did not markedly limit the expansion of *Escherichia–Shigella*, it still promoted the growth of *Bacteroides* to some extent. This pattern suggests that iron coordination altered polysaccharide fermentability and microbial accessibility, thereby redirecting community assembly toward polysaccharide-degrading taxa rather than broadly promoting opportunistic bacteria, as observed with free iron. LEfSe analysis (Fig. 3C-3E) further corroborated this distinction, showing preferential enrichment of *Lactobacillus* in GLPs groups and *Bacteroides* (mainly beneficial species including *Bacteroides vulgatus* and *Bacteroides caecimuris*) in GLPs–Iron complex groups (Fan et al., 2023; L. Liu et al., 2022; M. Xu et al., 2023; Z. Xu et al., 2025). Fermentation pH measurements (Fig. S2, Supplementary Materials) further supported the microbiota analysis, showing a significant pH decrease in the GLPs and GLPs–Iron complex groups compared with the CTL and Fe groups. This reduction in pH suggests enhanced microbial fermentation activity, which is commonly associated with the production of organic acids and is consistent with the enrichment of acid-producing bacteria such as *Lactobacillus* and *Bacteroides* (Flint et al., 2012). These results demonstrate that polysaccharide fractionation and iron coordination selectively modulate gut microbiota, promoting beneficial taxa while mitigating the expansion of opportunistic species.

## 4 Conclusion

Graded ethanol fractionation of *Glehnia littoralis* polysaccharides enabled the formation of polysaccharide–iron complexes with controlled iron coordination and preserved polysaccharide backbone. These complexes exhibited molecular weight–dependent iron release under simulated gastrointestinal conditions. Importantly, while free iron may disrupt gut microbiota, GLPs and GLPs–Iron complexes showed distinct modulatory effects on microbial communities during *in vitro* fermentation: native GLPs promoted *Lactobacillus* growth, whereas GLPs–Iron complexes favored beneficial *Bacteroides*. This work demonstrates that polysaccharide–Iron conjugation not only stabilizes iron for food applications but also provides microbiota-compatible benefits, offering a promising strategy for functional iron fortification.

## CRediT authorship contribution statement

**Xuan Hu:** Writing – original draft, Investigation, Data curation. **Xia Liu:** Investigation, Formal analysis, Data curation. **Yu Zhang:** Methodology, Investigation. **Yanli Yu:** Methodology. **Quanfang Zhang:** Investigation. **Xueyan Gao:** Methodology, Investigation, Data curation. **Wei Liu:** Writing – review & editing, Writing – original draft, Validation, Supervision, Methodology, Conceptualization.

## Declaration of competing interest

The authors declare that they have no known competing financial interests or personal relationships that could have appeared to influence the work reported in this paper.

## Data Availability

Data will be made available on request.

## Appendix A Supplementary data

Supplementary Materials. Image 1
