# Supplementary material for: Digestive behavior and gut microbiota responses of Glehnia littoralis polysaccharide–Iron complexes: Influence of polysaccharide molecular weight
Source: Food Chem X. 2026 Mar 20;35:103777. doi: 10.1016/j.fochx.2026.103777 (PMC13049535; doi:10.1016/j.fochx.2026.103777)
Supplement: Supplementary file 1 — Supplementary Materials. [file mmc1.docx]

**Digestive Behavior and Gut Microbiota Responses of *Glehnia littoralis* Polysaccharide–Iron Complexes: Influence of Polysaccharide Molecular Weight**

Xuan Hu^#1,2,3^, Xia Liu ^#1^, Yu Zhang^1,4^, Yanli Yu^3^, Quanfang Zhang^1^, Xueyan Gao*^2^, Wei Liu*^1^.

^1^ Institute of Food & Nutrition Science and Technology, Shandong Academy of Agricultural Sciences, Jinan 250100, China

^2^ Medical Science and Technology Innovation Center, Shandong First Medical University & Shandong Academy of Medical Sciences, Jinan 250117, China

^3^ Sports & Medicine Integration Research Center (SMIRC) Capital University of Physical Education and Sports, Beijing 100191, China

^4^ Institute of Resources and Environment, Dezhou Academy of Agricultural Sciences, Dezhou 253000, China

***Correspondence to:** Wei Liu, Email: 980701611@qq.com; Xueyan Gao, Email: gaoxueyan@sdfmu.edu.cn.

**#** These authors contributed equally to this work

**Key words:** *Glehnia littoralis*, Polysaccharide–Iron Complexes, In vitro digestion, In vitro fermentation, Gut microbiota.

**Table S1** Composition of simulated gastric fluid (SGF) and simulated intestinal fluid (SIF)

| **Component** | **concentration in SGF (mM)** | **concentration in SIF (mM)** |
| --- | --- | --- |
| **KCl** | 6.9 | 6.8 |
| **KH_2_PO_4_** | 0.9 | 0.8 |
| **NaHCO_3_** | 25 | 85 |
| **NaCl** | 47.2 | 38.4 |
| **MgCl_2_(H_2_O)_6_** | 0.12 | 0.33 |
| **(NH_4_)_2_CO_3_** | 0.5 | – |
| **HCl** | 15.6 | 8.4 |
| **CaCl_2_(H_2_O)_2_** | 0.15 | 0.6 |

**Table S2** Kinetic parameters of iron gastric release from GLPs–Iron complexes obtained by exponential model fitting

| $\boldsymbol{y=}\boldsymbol{F}_{\boldsymbol{max}}\boldsymbol{\times(1-}\boldsymbol{e}^{\boldsymbol{-kx}}\boldsymbol{)}$ | | | | |
| --- | --- | --- | --- | --- |
|  | **F_max_** | ***k*** | **AUC** | **R^2^** |
| P40Fe | 4.83% | 0.0261min^−1^ | 345.7%⋅min | 0.925 |
| P60Fe | 8.06% | 0.0279min^−1^ | 666.9%⋅min | 0.957 |
| P80Fe | 9.49% | 0.0248min^−1^ | 784.5%⋅min | 0.928 |

**Fig. S1**

**
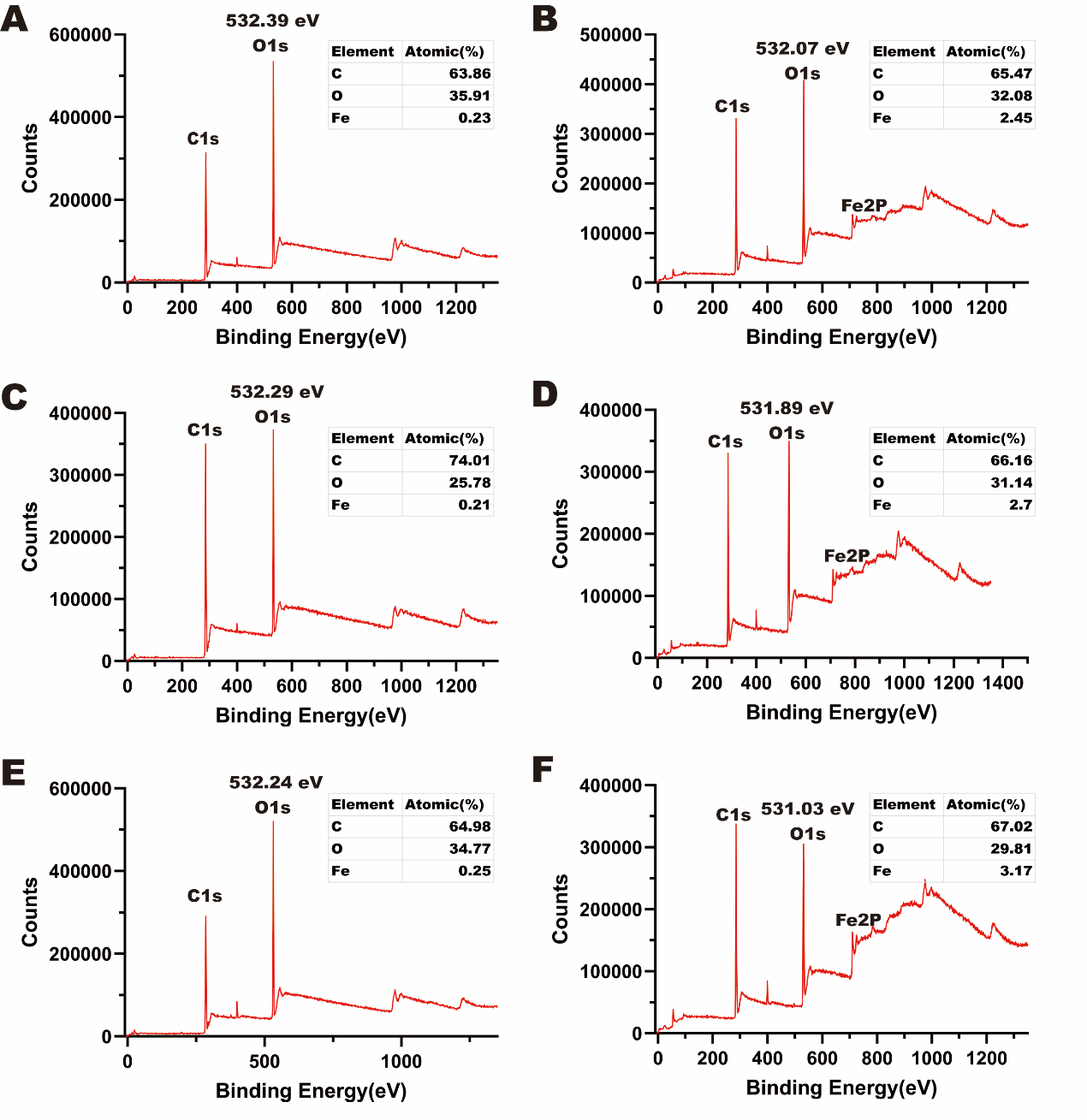
**

**Fig. S1.** X-ray photoelectron spectroscopy (XPS) analysis of GLPs and GLPs–Iron complexes. (A) P40; (B) P40Fe; (C) P60; (D) P60Fe; (E) P80; (F) P80Fe.

**Fig. S2**


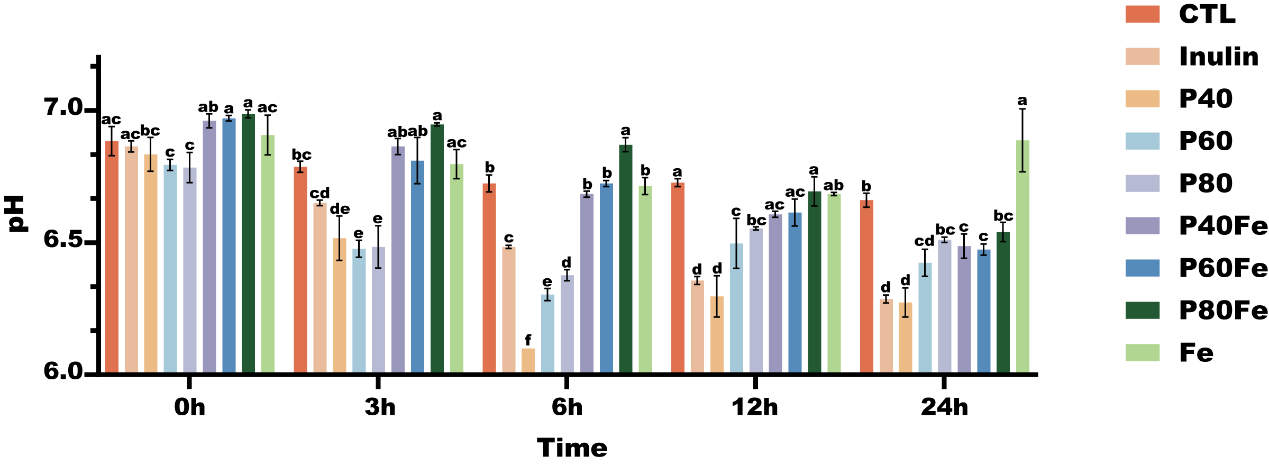


**Fig.S2.** Changes in pH value of the fermentation system during *in vitro* simulated fermentation process.
